# Effect of Gel Structure on the In Vitro Gastrointestinal Digestion Behaviour of Whey Protein Emulsion Gels and the Bioaccessibility of Capsaicinoids

**DOI:** 10.3390/molecules26051379

**Published:** 2021-03-04

**Authors:** Nan Luo, Aiqian Ye, Frances M. Wolber, Harjinder Singh

**Affiliations:** 1 Riddet Institute, Massey University, Private Bag 11 222, Palmerston North 4442, New Zealand; N.Luo@massey.ac.nz; 2 School of Food and Advanced Technology, Massey University, Private Bag 11 222, Palmerston North 4442, New Zealand; F.M.Wolber@massey.ac.nz

**Keywords:** emulsion gel, capsaicinoid, whey protein, bioaccessibility, in vitro dynamic digestion

## Abstract

This study investigated the effect of gel structure on the digestion of heat-set whey protein emulsion gels containing capsaicinoids (CAP), including the bioaccessibility of CAP. Upon heat treatment at 90 °C, whey protein emulsion gels containing CAP (10 wt% whey protein isolate, 20 wt% soybean oil, 0.02 wt% CAP) with different structures and gel mechanical strengths were formed by varying ionic strength. The hard gel (i.e., oil droplet size *d*_4,3_ ~ 0.5 μm, 200 mM NaCl), with compact particulate gel structure, led to slower disintegration of the gel particles and slower hydrolysis of the whey proteins during gastric digestion compared with the soft gel (i.e., *d*_4,3_ ~ 0.5 μm, 10 mM NaCl). The oil droplets started to coalesce after 60 min of gastric digestion in the soft gel, whereas minor oil droplet coalescence was observed for the hard gel at the end of the gastric digestion. In general, during intestinal digestion, the gastric digesta from the hard gel was disintegrated more slowly than that from the soft gel. A power-law fit between the bioaccessibility of CAP (Y) and the extent of lipid digestion (X) was established: Y = 49.2 × (X − 305.3)^0.104^, with *R*^2^ = 0.84. A greater extent of lipid digestion would lead to greater release of CAP from the food matrix; also, more lipolytic products would be produced and would participate in micelle formation, which would help to solubilize the released CAP and therefore result in their higher bioaccessibility.

## 1. Introduction

Emulsion-based systems for the delivery of lipophilic bioactive compounds, such as capsaicin, β-carotene and curcumin, by incorporating the bioactive compounds in the emulsion droplets, have been widely studied for the purposes of improving water solubility, stability and bioaccessibility [1,2,3,4,5]. For instance, Lu et al. [4] investigated the use of nanoemulsions for the delivery of capsaicin and reported that the bioaccessibility of capsaicin increased from about 10% in the unformulated form to about 80% in the capsaicin-loaded nanoemulsion after in vitro lipid digestion. However, the digestion conditions used in their study were very simple and did not represent gastrointestinal conditions. In our study, we used a Human Gastric Simulator to conduct in vitro dynamic digestion, which has been shown to better mimic the in vivo gastric conditions.

The behaviour of liquid emulsions during in vitro gastrointestinal digestion has been widely investigated in recent decades, one of the common behaviours investigated is flocculation/coalescence of oil droplets in the gastrointestinal tract [6,7,8]. Different from liquids, solid/semi-solid foods undergo more complicated processes during digestion, because they require mastication during oral processing, trituration and disintegration of the solid particles in the stomach to form particles that are small enough to pass through the pyloric sphincter. Therefore, solid/semi-solid foods usually take a longer time to digest and empty from the stomach and their digestion behaviour would be expected to affect the release behaviour of the nutrients and their bioaccessibility [9].

Emulsion gels have been widely used as a model system for solid/semi-solid foods, to investigate their structure, rheology and breakdown behaviour during digestion [10,11,12,13,14,15,16,17]. The structure and the rheological properties of the emulsion gel can be tailored so that its disintegration and digestion behaviour are altered [13,14,15]. Macierzanka et al. [18] studied the effect of protein structure on the kinetics of the simulated gastrointestinal digestion of bovine β-lactoglobulin (β-lg) by varying the pH or heating conditions under which the protein gel was formed; they reported that the gel that formed near the isoelectric point was most resistant to protein hydrolysis during simulated gastric digestion. Guo et al. [14] also reported that a whey protein emulsion gel containing 200 mM NaCl was disintegrated much more slowly during in vitro gastric digestion than a whey protein emulsion gel containing 10 mM NaCl. However, there is limited information on the use of emulsion-gel-based systems for the delivery of bioactive compounds and how the gel structure and the digestion behaviour affect their bioaccessibility.

Gastric emptying into the small intestine is a dynamic and continuous process, and is controlled by the pyloric sphincter. The food chyme leaving the stomach at different digestion times has distinct characteristics, which may have an impact on the bioaccessibility of the nutrients. However, little information on how the characteristics of the gastric chyme affects its behaviour during intestinal digestion and the bioaccessibility of nutrients is available. Therefore, in the present work, we collected gastric digesta from three different digestion times, to represent gastric digestion at the beginning, intermediate and final phases, and investigated how the characteristics of the gastric digesta affected the intestinal digestion and the bioaccessibility of capsaicinoids (CAP).

We used a whey protein emulsion gel as a model for solid/semi-solid delivery system; the capsaicinoids (CAP), i.e., the lipophilic bioactive compounds present in many peppers from the genus *Capsicum*, were dissolved in the emulsion droplets. The gel structure was modified by changing the NaCl concentration of the emulsion gel, so that the effect of gel structure on the disintegration and digestion behaviour of whey protein emulsion gels containing CAP could be studied.

## 2. Results and Discussion

Upon heat treatment at 90 °C, whey protein emulsion gels containing CAP (10 wt% whey protein isolate, 20 wt% soybean oil, 0.02 wt% CAP) with different gel structures and mechanical strengths were formed by varying ionic strength, as described previously [14,19]. A “hard gel” was formed at high ionic strength (i.e., 200 mM NaCl) and a “soft gel” at low ionic strength (i.e., 10 mM NaCl). In vitro masticated gel boluses from the soft gel and the hard gel were produced, which have been shown to have similar bolus particle sizes to the in vivo masticated gel boluses [19]. In vitro masticated gel boluses were used for simulated gastrointestinal digestion.

### 2.1. Physicochemical Characteristics of Emptied Gastric Digesta

#### 2.1.1. pH Changes during In Vitro Gastric Digestion

**Figure 1:**
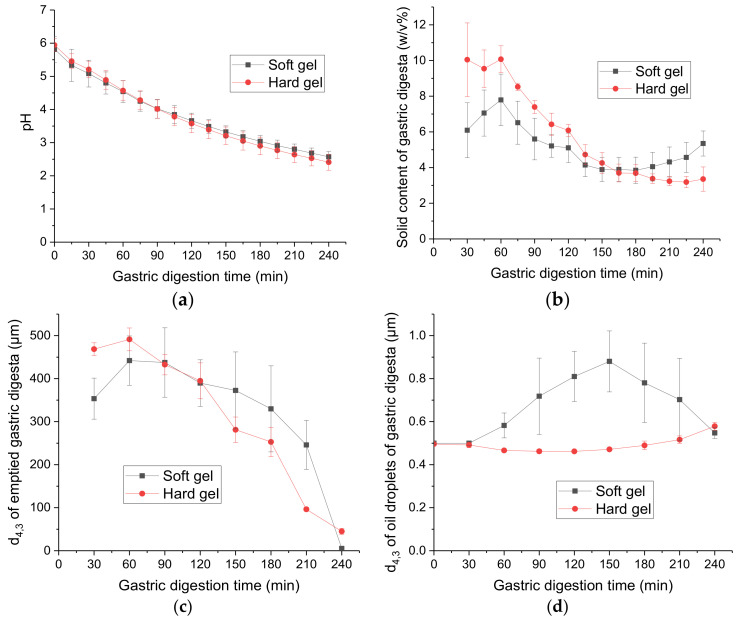
Physicochemical characteristics of the emptied gastric digesta of capsaicinoids (CAP)-loaded whey protein emulsion gels as a function of the digestion time: (**a**) pH; (**b**) solid content; (**c**) average particle size; (**d**) particle size of the oil droplets. Error bars represent standard deviations obtained from three replicates.

During 240 min of in vitro gastric digestion, with the constant addition of simulated gastric fluid (SGF, pH 1.5; secretion rate: 2.5 mL/min) and gastric emptying (emptying rate: 3 mL/min), the pH of the emptied gastric digesta, which essentially represents the pH of the gastric content, gradually decreased from 5.82 ± 0.39 at 0 min to 2.57 ± 0.16 at 240 min for the soft gel (i.e., CAP-loaded whey protein emulsion gel, oil droplet size *d*_4,3_ ~ 0.5 μm, 10 mM NaCl) and from 5.94 ± 0.20 at 0 min to 2.41 ± 0.24 at 240 min for the hard gel (i.e., CAP-loaded whey protein emulsion gel, *d*_4,3_ ~ 0.5 μm, 200 mM NaCl), as shown in Figure 1a. The trends of the pH profiles during gastric digestion were similar for both gels, indicating that the structure of the protein matrix did not have a significant effect on the buffering capacity of the emulsion gel.

The pH profiles were in agreement with an in vivo study reported by Kalantzi et al. [20], in which human participants were fed with 500 mL of Ensure Plus^®^ (a complete nutrient drink with a protein content of 62 mg/mL, a fat content of 49.2 mg/mL and a carbohydrates content of 202 mg/mL) and the pH of the gastric content was measured every 30 min for 3.5 h. An average pH of 2.7 was reported after 210 min of ingestion, which is comparable with the results obtained from the present work (pH ~ 2.80 ± 0.16 for the soft gel and pH ~ 2.64 ± 0.24 for the hard gel at 210 min of digestion).

#### 2.1.2. Solid Content of Emptied Gastric Digesta

Figure 1b shows the solid content of the emptied gastric digesta as a function of the digestion time. There were no data for 0 and 15 min because gastric emptying of solid foods starts only after 30 min of ingestion [21,22]. During the first 90 min of digestion, the solid content of the emptied gastric digesta from the hard gel remained higher than the soft gel (*P* < 0.05), suggesting that the hard gel was emptied out faster at the beginning of digestion. This could be attributed to the fact that the hard gel had a significantly smaller initial bolus particle size entering the stomach. According to the results obtained in our previous in vivo oral processing study [19], the masticated bolus particle size *d_frag_* was 0.8 mm for the hard gel and 1.7 mm for the soft gel. During 90 to 180 min of digestion, there was no significant difference in the solid content of the emptied gastric digesta from the two gels; it decreased gradually for both gels, because of the dilution of the gastric contents by constant gastric secretion and emptying. During 180 to 240 min of digestion, the solid contents of the emptied gastric digesta gradually increased for the soft gel whereas it remained relatively constant for the hard gel. This indicated that, towards the end of digestion, the soft gel was disintegrated and emptied out faster than the hard gel.

#### 2.1.3. Average Particle Size of Gel Particles in Emptied Gastric Digesta

The changes in the weight-to-volume diameter (*d*_4,3_, μm) of the emptied gastric digesta as a function of the digestion time are shown in Figure 1c. Overall, the average particle size of the emptied gastric digesta gradually decreased with increasing digestion time for both gels, indicating disintegration of the gel particles during gastric digestion. At 30 min (i.e., the time point for the first gastric emptying), the emptied gastric digesta from the hard gel had a significantly larger particle size than that from the soft gel. The human gastric sieving, mimicked using a mesh bag, allowed only particles smaller than 1 mm to pass through and empty out. During oral processing, the soft gel was mainly fragmented into bigger particles, and about 75% of the masticated gel bolus was larger than 1.0 mm, which could not be emptied out at the beginning of gastric digestion. In contrast, the hard gel was fragmented into smaller particles during mastication, and about 50% of the masticated gel bolus was smaller than 1.0 mm [19].

During the first 90 min of digestion, the soft gel showed an increasing trend in the *d*_4,3_ of the emptied gastric digesta, whereas the hard gel showed a slight decreasing trend. This could have been because of higher swelling of the soft gel. Guo [23] reported that a soft gel (whey protein emulsion gel containing 10 mM NaCl) had a significantly higher swelling ratio in simulated gastric fluid (SGF) (pH 1.5) than a hard gel (whey protein emulsion gel containing 200 mM NaCl). Although the swelling ratio gradually increased with increasing incubation time in the SGF for both gels, after 4 h of incubation, the swelling was about 10% for the soft gel and about 2% for the hard gel. It could also have been that gel particles from the soft gel that were bigger than 1 mm gradually disintegrated and were emptied out.

During 90 to 240 min of digestion, the *d*_4,3_ of the emptied gastric digesta gradually decreased for both gels; the decrease was especially noticeable after 180 min, when the gastric pH dropped to below 3.0 and the gel particles were rapidly disintegrated.

#### 2.1.4. Size of Oil Droplets in Emptied Gastric Digesta

The *d*_4,3_ of the oil droplets in the emptied gastric digesta as a function of digestion time is presented in Figure 1d. The oil droplet size of the emptied gastric digesta from the hard gel increased slightly after 210 min of digestion, indicating minor coalescence of the oil droplets at the end of digestion. For the soft gel, the oil droplet size increased between 60 and 150 min of digestion, indicating that the oil droplets started to be released from the gel matrix at 60 min and underwent coalescence, possibly because of hydrolysis of adsorbed proteins by pepsin. The average oil droplet size showed a decreasing trend between 150 and 240 min of digestion, possibly because of gradual creaming of the coalesced oil droplets inside the Human Gastric Simulator (HGS).

### 2.2. Microstructure of Emptied Gastric Digesta

**Figure 2:**
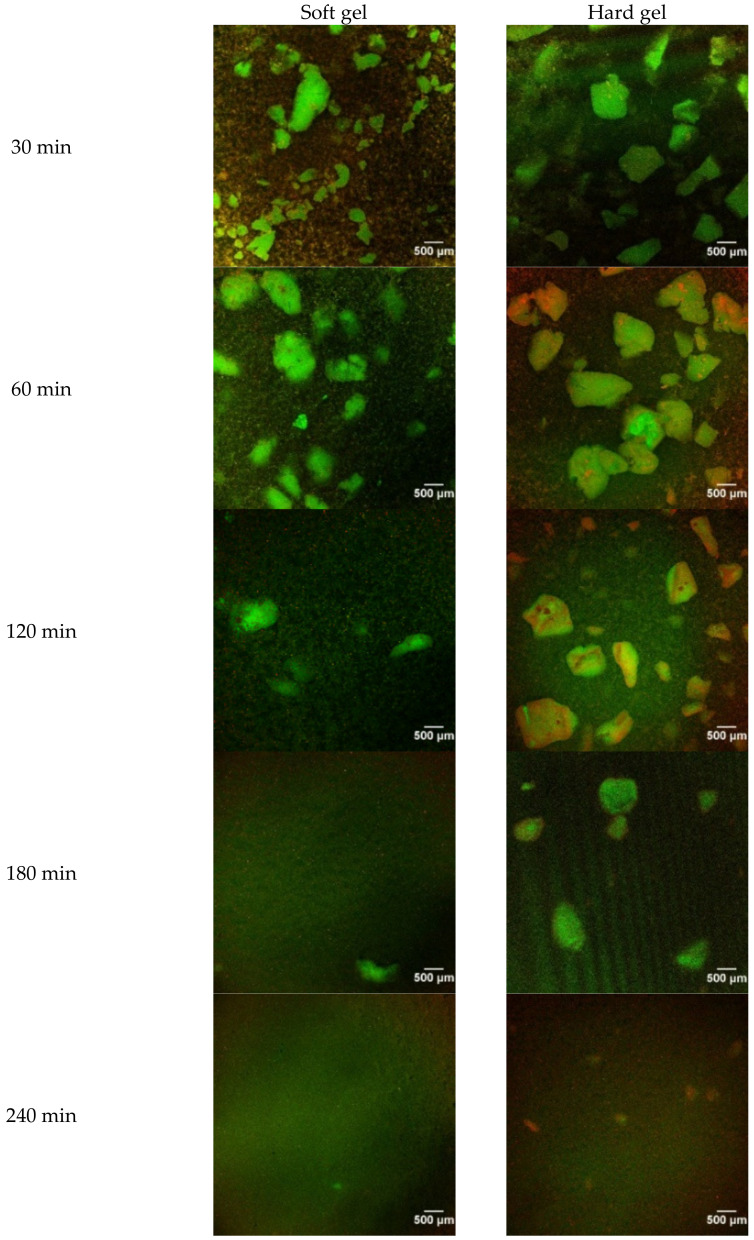
Confocal laser scanning microscopy (CLSM) images of the gastric digesta as a function of digestion time.

Confocal laser scanning microscopy (CLSM) images of the emptied gastric digesta showed gradual disintegration of the gel particles with increasing digestion time for both gels Figure 2. Disintegration of the gel particles was not obvious for the hard gel in the first 120 min of digestion, which is consistent with the results presented in Figure 1c. For the soft gel, at 120 min of digestion, many large gel particles (~500 μm) in the digesta disappeared and disintegrated into smaller particles. At 240 min of digestion, gel particles of ~200 μm in size were still observed in the digesta from the hard gel, whereas the soft gel seemed to be mainly broken down into particles smaller than 100 μm. The CLSM images indicate that the soft gel was disintegrated much faster than the hard gel during gastric digestion.

### 2.3. SDS-PAGE Patterns of Emptied Gastric Digesta

**Figure 3:**
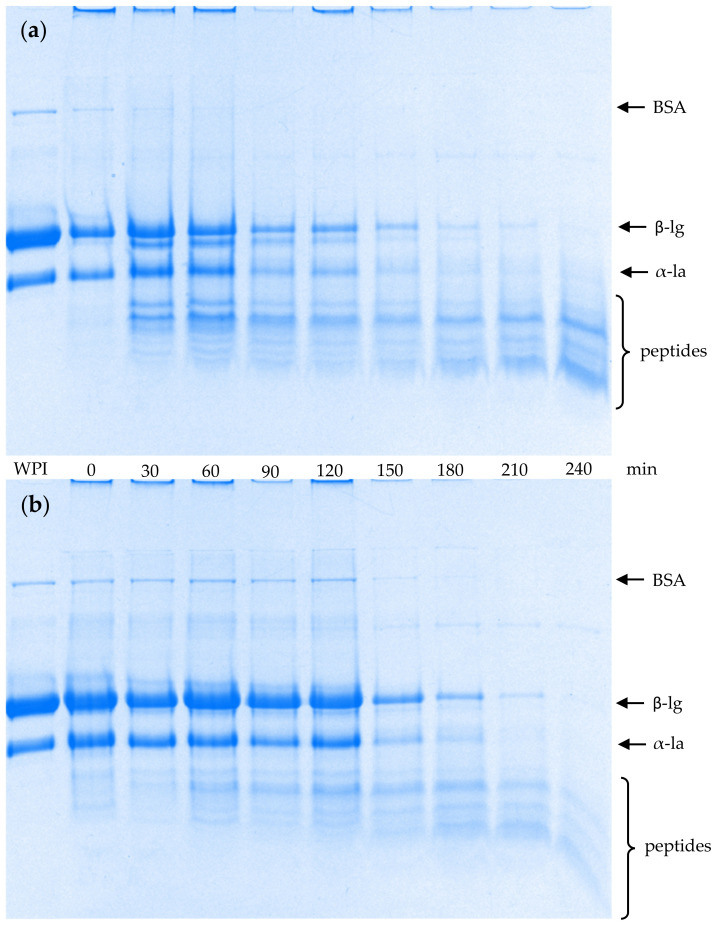
Tricine sodium dodecyl sulphate polyacrylamide gel electrophoresis patterns under reducing conditions of emptied gastric digesta as a function of digestion time: (**a**) soft gel; (**b**) hard gel. WPI: whey protein isolate; BSA: bovine serum albumin; β-lg: β-lactoglobulin; α-la: α-lactalbumin.

Figure 3 shows the sodium dodecyl sulphate polyacrylamide gel electrophoresis (SDS-PAGE) patterns under reducing conditions of the emptied gastric digesta as a function of digestion time. The bands from both gels consisted mainly of bovine serum albumin (BSA), β-lg and α-lactalbumin (α-la). Peptide bands appeared after 30 min of digestion for both gels, indicating protein hydrolysis by pepsin. However, the intensities of the peptide bands at 30 to 210 min of digestion were much lower from the hard gel than from the soft gel, whereas the intensities of the BSA, β-lg and α-la bands were more intense, even though the solid content of the digesta from the hard gel was significantly lower than that from the soft gel at 30, 60 and 90 min of digestion but was similar at 120, 150, 180 and 210 min of digestion. This indicates that the whey proteins were hydrolysed more slowly in the hard gel than the soft gel. The result is in agreement with the reports from Guo et al. [14] and Liang, Leung Sok Line, Remondetto, and Subirade [24], in which it was stated that β-lg degraded more rapidly when the β-lg stabilized emulsion gel had a filamentous structure (soft gel) than when it had a particulate structure (hard gel).

The hard gel has a more compact particulate gel structure and there is more cross-linking between the whey proteins within the gel [18,25,26,27]. Consequently, some cleavage sites may be changed and become less accessible for pepsin to approach during gastric digestion, leading to slower hydrolysis of the whey proteins. Even though the whey proteins in both gels started to be hydrolysed by pepsin at approximately the same time, the rate of proteolysis was slower in the hard gel. The faster proteolysis and disintegration of the soft gel may have caused the earlier and more severe occurrence of oil droplet coalescence in the gel particles from the soft gel Figure 1d.

A key factor contributing to the slower proteolysis and disintegration of the hard gel could have been the slower diffusion rate of pepsin in the gel particles of the hard gel. Somaratne et al. [28] studied the pepsin diffusivity in relation to the microstructure of an egg white gel and found that a gel with a compact and homogeneous microstructure had a lower diffusion coefficient than a gel with a heterogeneously loose protein matrix with large aggregate particles.

There were no intact whey proteins remaining after 240 min of digestion, suggesting that, even though proteolysis was slowed in the hard gel, the cleavage sites may have been more difficult to access by pepsin but were not hindered.

### 2.4. In Vitro Intestinal Digestion

The gastric digesta emptied at 60, 120 and 240 min of digestion were used for in vitro intestinal digestion because these time points can represent gastric digestion at three different phases: beginning, intermediate and final phases. Gastric digesta collected at these time points had different compositions and properties.

The fat contents for the gastric digesta emptied at 60, 120 and 240 min were 4.0 ± 0.7, 2.3 ± 0.2 and 3.1 ± 0.4 *w/v*% for the soft gel and 5.6 ± 0.5, 3.2 ± 0.4 and 2.0 ± 0.5 *w/v*% for the hard gel.

#### 2.4.1. Breakdown of Gel Particles during Intestinal Digestion

**Figure 4:**
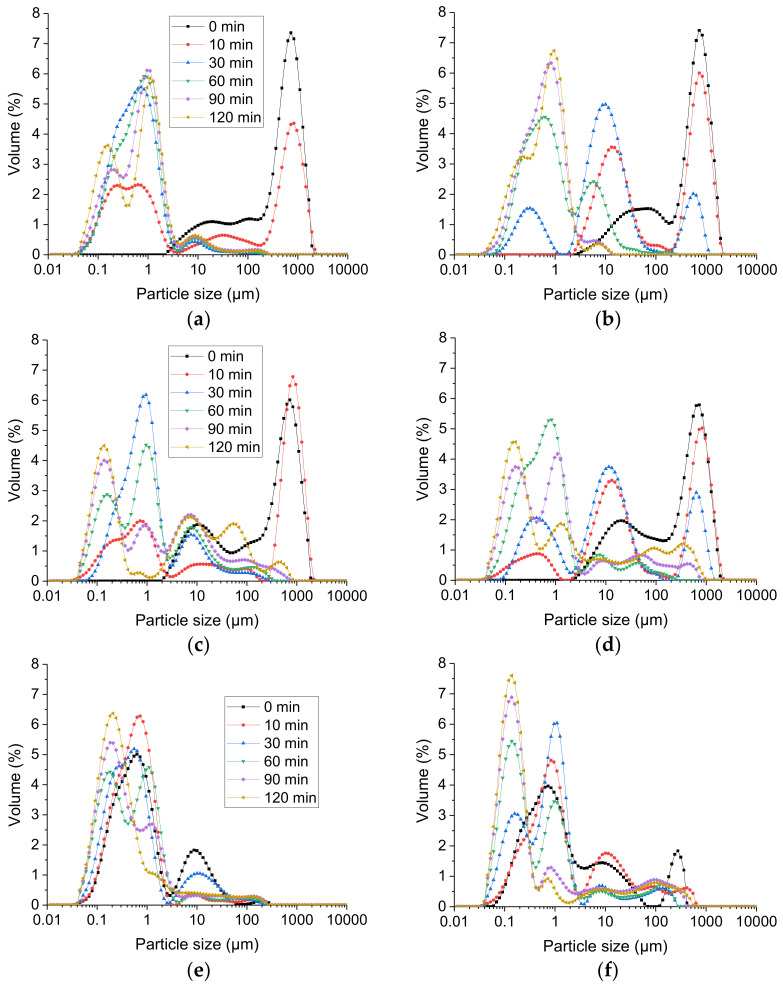
Changes in particle size distributions of digesta during 120 min of intestinal digestion (0, 10, 30, 60, 90 and 120 min). Gastric digesta emptied at different gastric digestion times from the soft gel and the hard gel were used for intestinal digestion: (**a**) gastric digesta from the soft gel emptied at 60 min; (**b**) gastric digesta from the hard gel emptied at 60 min; (**c**) gastric digesta from the soft gel emptied at 120 min; (**d**) gastric digesta from the hard gel emptied at 120 min; (**e**) gastric digesta from the soft gel emptied at 240 min; (**f**) gastric digesta from the hard gel emptied at 240 min.

Figure 4 presents the changes in particle size distribution during in vitro intestinal digestion of the gastric digesta emptied at 60, 120 and 240 min of gastric digestion from the two gels. The gastric digesta emptied at 60 min from both gels had similar particle size distributions, showing a bimodal pattern with a narrow peak near 1000 μm and another broader peak in the range 2–100 μm. For both gels, there was no peak in the range 0.1–1 μm, indicating no oil droplet release from the protein matrix Figure 4a,b. At 10 min of intestinal digestion, the digesta from the hard gel still showed a bimodal distribution, in which the peak near 10 μm became narrower and increased in volume whereas the peak near 1000 μm decreased slightly in volume, indicating disintegration of the gel particles. No free oil droplets were released from the protein matrix at this stage. The digesta from the soft gel showed a trimodal distribution, with a new peak appearing in the range 0.04–4 μm, indicating the liberation of free oil droplets from the protein matrix at 10 min of intestinal digestion; the peaks in the range 2–100 μm and near 1000 μm both decreased in volume, indicating disintegration of the gel particles. At 30 min of intestinal digestion, the digesta from the hard gel showed a trimodal distribution with a new peak appearing in the range 0.05–2 μm, suggesting oil droplet release from the protein matrix; the peak near 10 μm increased in volume and the peak near 1000 μm decreased in volume. For the digesta from the soft gel, the peak in the range 0.04–4 μm continued to increase in volume whereas that in the range 4–300 μm decreased in volume, indicating further breakdown of the gel particles and more oil droplet release at 30 min of intestinal digestion.

At 60, 90 and 120 min of intestinal digestion, the particle size distributions of the digesta from the soft gel were similar, with a major peak in the range 0.04–4 μm and another small peak with a tail in the range 4–300 μm. The peak in the range 0.04–4 μm slowly transformed into a bimodal pattern with a peak near 0.1 μm and another peak near 1 μm. The peak near 0.1 μm may have been mixed micelles and vesicles formed during the intestinal digestion or the digested oil droplets; that near 1 μm may have been undigested oil droplets. The peak with a tail in the range 4–300 μm may have been coalesced/flocculated oil droplets. At 60, 90 and 120 min of intestinal digestion, the digesta from the hard gel also showed a bimodal distribution, with a major peak in the range 0.04–4 μm and a second peak in the range 4–100 μm. The peak in the range 4–100 μm gradually decreased in volume from 60 to 120 min of intestinal digestion; that in the range 0.04–4 μm gradually increased in volume and showed a bimodal distribution at 120 min of digestion; however, the peak near 0.1 μm was much smaller than that from the soft gel. This qualitatively indicates that the soft gel was digested to a greater extent.

The changes in particle size distributions during the intestinal digestion of the gastric digesta emptied at 120 min are shown in Figure 4c (soft gel) and Figure 4d (hard gel). The particle size distributions of the digesta from both gels before intestinal digestion (i.e., 0 min) were similar, with a peak near 1000 μm and another peak in the range 2–200 μm. There was no peak in the range 0.1–1 μm, which possibly meant that there was no oil droplet release from the protein matrix. At 10 min of intestinal digestion, for the digesta from both gels, a new peak in the range 0.03–2 μm appeared, indicating the release of oil droplets from the protein matrix; the peak near 1000 μm was similar to that of the digesta at 0 min. The peak in the range 2–200 μm decreased in the digesta from the soft gel and increased in the digesta from the hard gel. At 30 min of intestinal digestion, the peak near 1000 μm had disappeared in the digesta from the soft gel, indicating the complete breakdown of the large gel particles from the soft gel, whereas the digesta from the hard gel still had this peak. In general, the peaks gradually shifted to the left for both gels, indicating particle size reduction during intestinal digestion. At 120 min of intestinal digestion, there was a major peak near 0.1 μm for both gels, indicating digested oil droplets or the formation of mixed micelles and vesicles, and some small peaks in the larger size ranges, which could have been undigested and/or coalesced/flocculated oil droplets.

The changes in the particle size distributions of the gastric digesta emptied at 240 min are shown in Figure 4e (soft gel) and Figure 4f (hard gel). At 0 min, the digesta from the soft gel showed a bimodal distribution, with one peak near 10 μm representing small gel particles and another peak near 0.5 μm representing free oil droplets released from the protein matrix; the digesta from the hard gel showed a trimodal distribution, with a peak near 300 μm and a peak near 10 μm representing gel particles and a third peak near 0.7 μm representing free oil droplets released from the protein matrix. Starting from 30 min of intestinal digestion, the digesta from both gels showed a bimodal distribution in the range 0.04–4 μm, with a peak near 0.1 μm and another peak near 1 μm. The peak near 0.1 μm gradually increased in volume and reached a maximum at the end of the intestinal digestion, whereas the peak near 1 μm gradually decreased in volume. This indicated the gradual digestion of oil droplets and the formation of mixed micelles and vesicles during the intestinal digestion.

In summary, the results indicate that the digesta from the hard gel emptied at 60 and 120 min were more resistant to disintegration and digestion in the small intestine than those from the soft gel, and this could be attributed to the higher solid content of the digesta from hard gel; the effect of the protein matrix is another contributing factor, as explained above.

In general, the gastric digesta emptied at 240 min were disintegrated the fastest in the small intestine, followed by those emptied at 120 min and those emptied at 60 min. This was probably because of the lower solid content and smaller average gel particle size of the gastric digesta emptied at the later digestion time.

#### 2.4.2. Free Fatty Acid Release Profile

**Figure 5:**
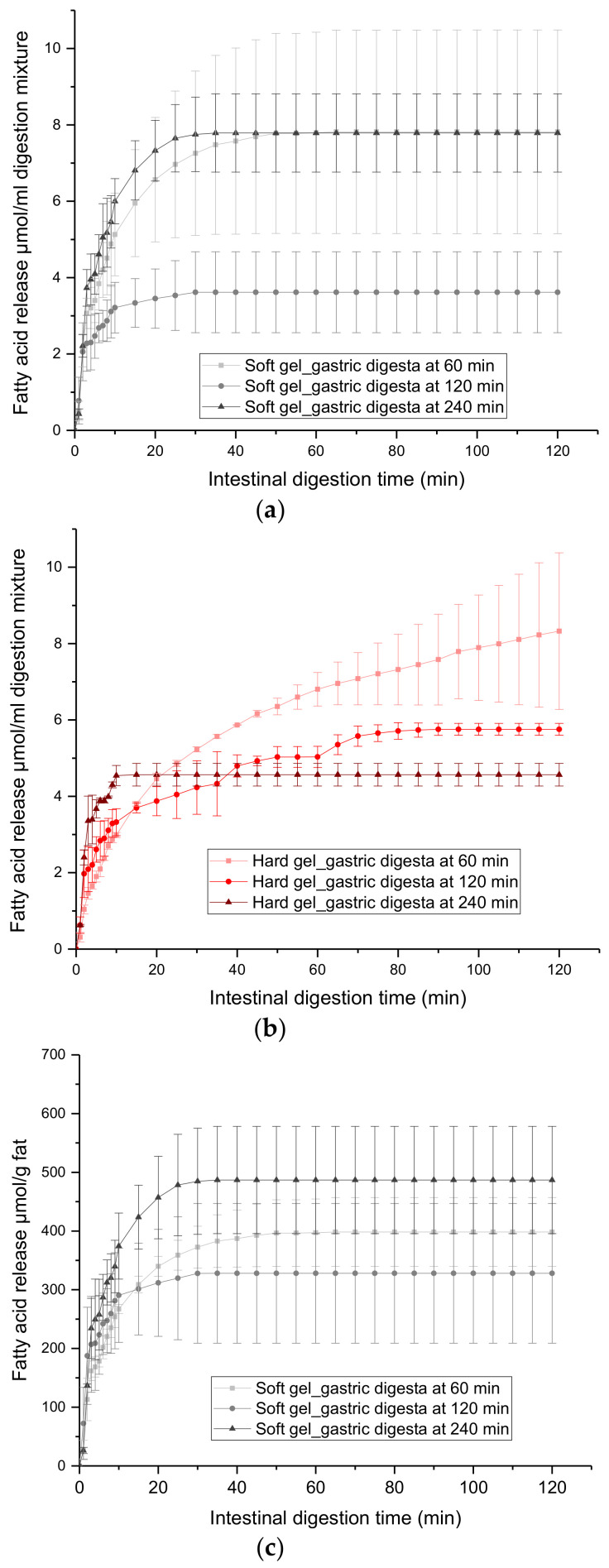

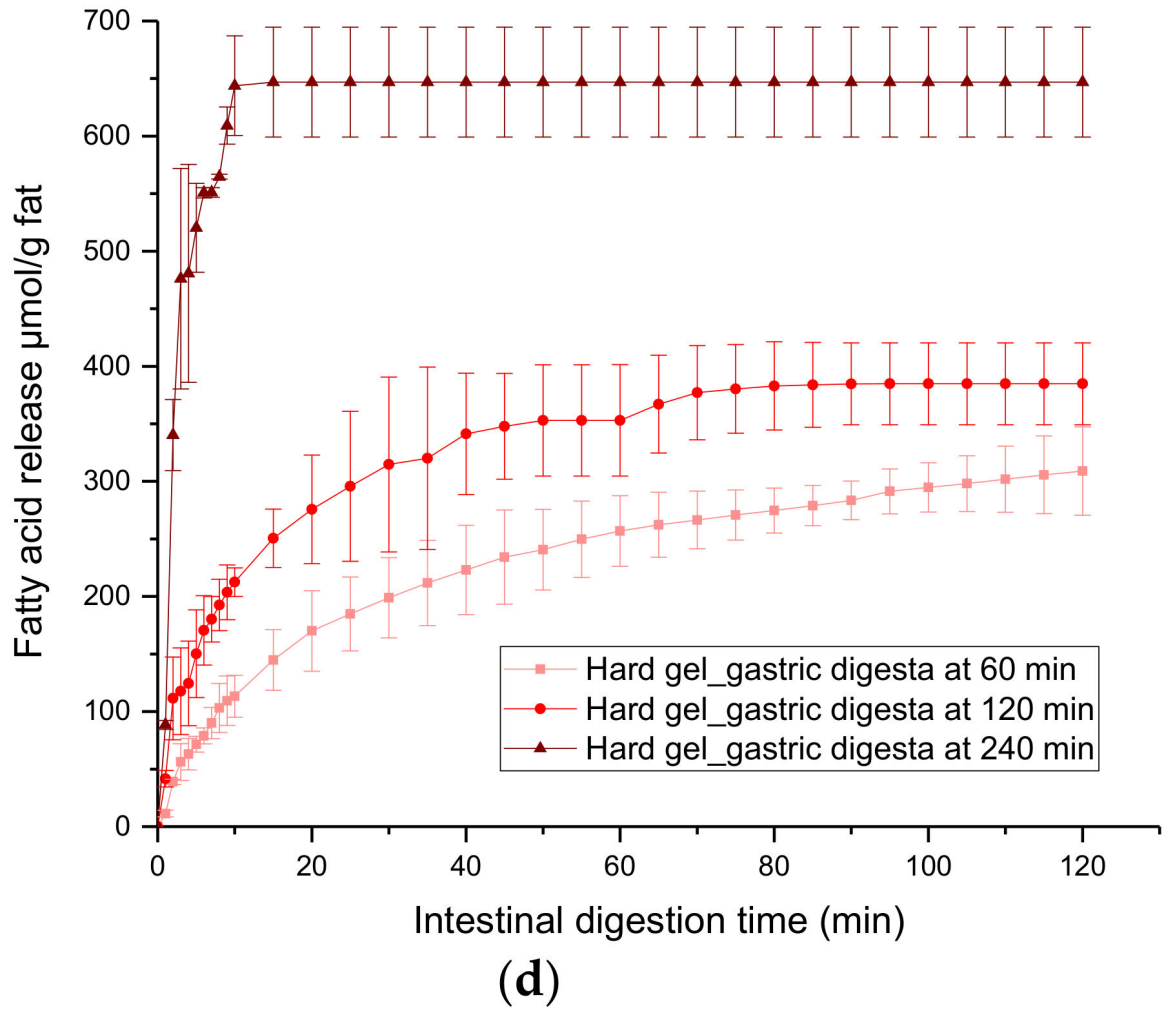
Free fatty acid release profile per mL of digestion mixture of gastric digesta emptied at 60, 120 and 240 min from: (**a**) soft gel, (**b**) hard gel. Free fatty acid release profile per gram of fat of gastric digesta emptied at 60, 120 and 240 min from: (**c**) soft gel; (**d**) hard gel. Error bars represent standard deviations obtained from three replicates.

The free fatty acid (FFA) release profiles per mL of digestion mixture of the gastric digesta emptied at 60, 120 and 240 min during 120 min of intestinal digestion are shown in Figure 5a (soft gel) and Figure 5b (hard gel). For the gastric digesta at 60 min, fatty acid release reached a plateau at about 50 min of intestinal digestion for the soft gel, whereas it had not reached a plateau at the end of the intestinal digestion for the hard gel. This indicated that the oil droplets were hydrolysed faster from the soft gel than the hard gel, and this could also be attributed to the effect of the gel structure. The oil droplets were liberated from the protein matrix more rapidly in the soft gel than in the hard gel [indicated by the particle size distributions presented in Figure 4a,b, which made it easier for the pancreatic lipase and co-lipase to access the interface of the oil droplets. Similar results were observed for the gastric digesta at 120 min. For the gastric digesta at 240 min, it took 30 min for the soft gel to reach a plateau whereas it took 10 min for the hard gel. This could be attributed to the lower fat content of the gastric digesta from the hard gel.

For the gastric digesta emptied at 60 min, the lipolysis rate (i.e., the slope of the curve) of the soft gel gradually decreased in the first 50 min of digestion because of gradual lipolysis of the oil droplets (i.e., a decreased substrate concentration leading to a decreased rate of reaction), and the rate became zero after 50 min of intestinal digestion. For the hard gel, the lipolysis rate decreased slightly in the first 30 min and then remained relatively constant until the end of digestion. This meant that, in the first 30 min, the concentration of accessible substrate gradually decreased, and that, subsequently, the ratio of accessible substrate to enzyme was relatively constant, indicating that the hard gel was broken down at a slower rate during intestinal digestion, leading to the gradual release of accessible oil droplets. Similar results were observed for the gastric digesta emptied at 120 min.

Figure 5c (soft gel) and Figure 5d (hard gel) show the free fatty acid release profiles per gram of fat, which indicates the extent of lipolysis during intestinal digestion. For the hard gel, the gastric digesta emptied at 240 min had the highest extent of lipolysis, followed by the gastric digesta emptied at 120 min, and then the gastric digesta emptied at 60 min (*P* < 0.05). This could be because the gastric digesta at 240 min had the least fat content and smallest particle size of the gel particles. For the soft gel, no significant difference was found in the extent of lipolysis between gastric digesta emptied at different gastric digestion times. This could also be attributed to the gel structure of the soft gel. The soft gel was digested more readily, and therefore, the differences in particle size or fat content between the gastric digesta emptied at different times did not lead to significant differences in the extent of lipolysis. For the gastric digesta emptied at 60 min, the soft gel had a greater extent of lipid digestion than the hard gel (*P* < 0.05); there was no significant difference between the soft gel and the hard gel for the gastric digesta emptied at 120 and 240 min.

The extent of lipid digestion is affected by multiple factors, such as the accessibility of lipolytic enzymes to the substrate, the concentration of the substrate, the concentrations of the enzyme and the bile salts, the effect of calcium, the characteristics of the lipids (e.g., type of lipid, droplet size, emulsifier type), etc. [5,29,30,31,32]. It is expected that a lower fat content would generally lead to a greater extent of lipid digestion [29], because of a higher ratio of calcium/bile salts to the substrate/FFAs. The role of bile salts in lipid digestion is that they replace the proteins/peptides at the oil–water interface, so that the co-lipase and lipase can anchor on to the interface and hydrolyse the lipids; bile salts also replace the lipolytic products (i.e., FFAs, monoglycerides and diglycerides) from the interface and help solubilize them by forming mixed micelles [32]. With a higher ratio of calcium/bile salts-to-substrate, the lipolytic products would be more effectively precipitated by calcium and displaced by bile salts from the oil–water interface. This would prevent the accumulation of lipolytic products at the interface, which would free the interface for the enzymes to further hydrolyse the lipid. The effect of lower fat content on the extent of lipid digestion was clearly noticeable: (1) for the hard gel, the gastric digesta emptied at 240 min had a significantly greater extent of lipid digestion than the gastric digesta emptied at 60 and 120 min; (2) for the gastric digesta emptied at 60 min, the soft gel had a greater extent of lipid digestion than the hard gel; (3) for the gastric digesta emptied at 240 min, the hard gel had a greater extent of lipid digestion than the soft gel.

However, for the gastric digesta emptied at 120 min from the soft gel, even though it had a lower fat content than the gastric digesta emptied at 60 and 240 min (*P* < 0.05), the extent of lipid digestion was not significantly different. At 120 min, it is likely that oil droplet coalescence had occurred, which increased the oil droplet size, compared with the gastric digesta emptied at 60 and 240 min Figure 1d. An increase in oil droplet size would lead to a decrease in the interface area exposed to lipolytic enzymes, and therefore to a decrease in the rate of lipid digestion [5,29].

#### 2.4.3. Initial Lipolysis Rate

The initial lipolysis rate (i.e., the FFAs released per mL of digestion mixture per minute during the initial 2 min of reaction) calculated from Figure 5a,b is presented in Table 1. For the gastric digesta emptied at 60 min, the soft gel had a higher lipolysis rate than the hard gel (*P* < 0.05); no significant difference between the soft gel and the hard gel was found for the gastric digesta emptied at 120 and 240 min. Based on the Michaelis–Menten equation, the rate of the enzymatic reaction is positively correlated with the substrate concentration until the reaction rate reaches its maximum value and plateaus (i.e., the enzyme becomes saturated). However, the limitation of the Michaelis–Menten model is that it assumes free diffusion. For the gastric digesta emptied at 60 min, the hard gel had a significantly higher fat content than the soft gel; however, the initial lipolysis rate was significantly lower. This suggests that the gel structure had a stronger effect on the lipolysis rate than the concentration of substrate (i.e., fat). As the oil droplets in the gel particles were covered by protein in both gels, the diffusion of enzymes through the solid gel particles would have been lower in the hard gel than in the soft gel.

For the gastric digesta emptied at 120 min, the hard gel had a significantly higher fat content than the soft gel, but there was no significant difference in the initial lipolysis rate. This could also be attributed to the effect of the gel structure of the soft gel. For the gastric digesta emptied at 240 min, the soft gel had a higher initial lipolysis rate than the hard gel. There could have been several contributing factors: the gastric digesta from the soft gel had a significantly higher fat content, smaller gel particle size, and more oil droplet release from the protein matrix. For both gels, the gastric digesta emptied at 240 min had a higher lipolysis rate than the gastric digesta emptied at 60 min. At 240 min of gastric digestion, the solid content was much lower, the gel particles were broken down into smaller sizes, and free oil droplets were released from the protein matrix. These factors would make the substrate easier for the enzyme to access. Additionally, the proteins in the gastric digesta at 240 min were more readily hydrolysed than the proteins in the gastric digesta at 60 min, which may also have contributed to the higher lipolysis rate of the gastric digesta at 240 min.

### 2.5. Bioaccessibility of CAP

**Figure 6:**
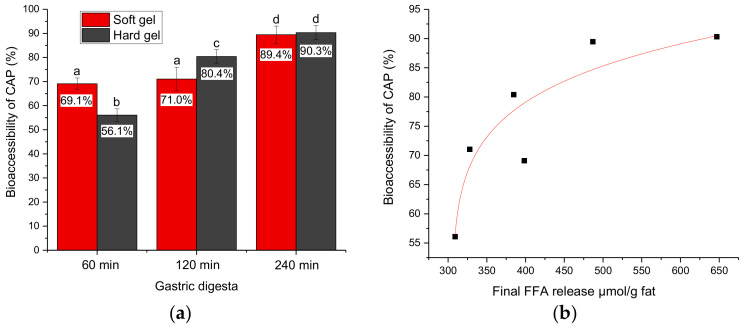
(**a**) Bioaccessibility of capsaicinoids (CAP) after in vitro gastrointestinal digestion. Error bars represent standard deviations obtained from three replicates. (**b**) Correlation between the bioaccessibility of CAP (Y) and the final free fatty acid (FFA) release per gram of fat (X): Y = 49.2 × (X − 305.3)^0.104^ *R*^2^ = 0.84.

Figure 6a presents the bioaccessibility of CAP after in vitro gastrointestinal digestion. For both gels, the gastric digesta emptied at 240 min had significantly higher bioaccessibility of CAP than the gastric digesta emptied at 60 and 120 min. For the gastric digesta emptied at 60 min, the soft gel had a higher bioaccessibility of CAP than the hard gel (*P* < 0.05); for the gastric digesta emptied at 120 min, the hard gel had a higher bioaccessibility of CAP than the soft gel (*P* < 0.05); there was no significant difference between the soft gel and the hard gel for the gastric digesta emptied at 240 min. A power-law fit between the bioaccessibility of CAP (Y) and the extent of lipid digestion (X; represented by the final FFA release per gram of fat) was established: Y = 49.2 × (X − 305.3)^0.104^, with *R*^2^ = 0.84 Figure 6b. In general, the bioaccessibility of CAP was positively correlated with the extent of lipid digestion.

The absorption of dietary CAP includes the following steps: the release of CAP from the food matrix during digestion in the gastrointestinal tract, the solubilization of CAP in the aqueous phase, the transportation of CAP to the epithelium and the absorption of CAP by the epithelial cells. In our in vitro studies, only the first two steps, which represent the bioaccessibility of CAP, were considered. A greater extent of lipid digestion would lead to greater release of CAP from the food matrix; also, more lipolytic products would participate in micelle formation and would help solubilize the released CAP, and therefore would lead to higher bioaccessibility. The results were in agreement with the report from Lu et al. [4]; they investigated the effect of different oil types (medium chain triglycerides, corn oil and canola oil) and found that the use of medium chain triglycerides as the lipid carrier for capsaicin in a nanoemulsion resulted in a significantly greater extent of lipolysis during in vitro digestion, and that this led to the greater bioaccessibility of capsaicin. In a study investigating the bioaccessibility of β-carotene in emulsions and nanoemulsions, Salvia-Trujillo et al. [5] also reported that the bioaccessibility of β-carotene and the final FFAs released were positively linearly correlated.

## 3. Materials and Methods

### 3.1. Materials

Powdered capsaicinoid (CAP) (61% capsaicin, 32% dihydrocapsaicin and 2.5% other capsaicinoids) was purchased from Wuxi AccoBio Biotech Inc., Wuxi, Jiangsu, China. Whey protein isolate 895, instantized and with 93% protein content (WPI), was purchased from Fonterra Co-operative Group Limited, Auckland, New Zealand. Soybean oil was purchased from Davis Trading Company, Palmerston North, New Zealand, and was used without further purification. Milli-Q water (Milli-pore Corp., Bedford, MA, USA) was used for all experiments. Pepsin from porcine gastric mucosa (#P7000: ≥250 units/mg solid), pancreatin from porcine pancreas (#P7545: 8 × USP), amano lipase A from *Aspergillus niger* (#534781: ≥12 000 U/g), bile bovine (#B3883) and Pefabloc^®^ SC (#76307) were purchased from Sigma-Aldrich (St. Louis, MO, USA). Other chemical reagents used in this study were of analytical grade and were used without further modification, unless otherwise specified.

Simulated salivary fluid (SSF), simulated gastric fluid (SGF) and simulated intestinal fluid (SIF) were prepared following the instructions from Minekus et al. [33] with slight modifications. The 1.25× concentrates of the simulated digestion fluids were referred to as stock simulated digestion fluids. The stock simulated digestion fluids were magnetically stirred at room temperature for 2 h to allow complete dissolution. The pHs of stock SSF (1.25×), stock SGF (1.25×) and stock SIF (1.25×) were adjusted to 7.0, 1.5 and 7.0, respectively, by 6 M HCl/10 M NaOH. The stock simulated digestion fluids were stored at 4 °C, warmed to room temperature in a water bath before use and used within one month after preparation.

### 3.2. Methods

#### 3.2.1. Preparation of CAP-Loaded Emulsion

Powdered CAP and WPI were added to soybean oil and water, respectively. The solutions were magnetically stirred for 4 h at room temperature to allow complete dissolution. A CAP-loaded coarse emulsion containing 0.02 wt% CAP, 19.98 wt% soybean oil and 10 wt% WPI was prepared using a rotor–stator Ultra-Turrax (LabServ D500, Thermo Electron LED GmBH, Langenselbold, Hessen, Germany) at 12,000 rev/min for 3 min. The coarse emulsion was then homogenized by passing it four times through a two-stage valve homogenizer (Homolab 2, FBF ITALIA SRL, Sala Baganza, Parma, Italy) at first/second stage pressures of 270/20 bar to generate an average oil droplet size (*d*_4,3_) of ~ 0.5 ± 0.05 μm.

#### 3.2.2. Formation of CAP-Loaded Emulsion Gels

The required quantities of NaCl were added to the CAP-loaded emulsion to give final concentrations of 10 mM and 200 mM NaCl, respectively. They were gently stirred for 1 h to allow complete dissolution of the NaCl. The emulsions were then poured into plastic tubes (inner diameter, 25 mm; capacity, 35 mL), sealed, heated in a water bath from 30 to 90 °C for 10 min and held at 90 °C for 20 min. The plastic tubes were immersed in an ice/water mixture immediately after heating and then stored at 4 °C overnight until further use. The CAP-loaded emulsion gels with *d*_4,3_ of ~ 0.5 ± 0.05 μm and containing 10 mM and 200 mM NaCl were referred to as the soft gel and the hard gel, respectively.

#### 3.2.3. Preparation of Simulated Masticated Gel Bolus

A food processer (The Mini Wizz food chopper, BFP100, Breville Group Ltd., Australia) was used to mimic oral breakdown of the CAP-loaded emulsion gels and to produce in vitro masticated gel boluses that had similar bolus particle sizes to in vivo masticated gel boluses [19]. The CAP-loaded emulsion gels were cut into cylinders of 12 mm in height and 25 mm in diameter, and seven cylindrical gel samples were added into the processer. The soft gel was ground for 4 s. The hard gel was initially ground for 3 s, and then a portion of ~10 g was taken out; the rest was ground for another 5 s, and then ~10 g was taken out; the remaining gel particles were ground for another 9 s; all three portions were then mixed together. Experiments were carried out at room temperature. Simulated masticated boluses of the soft gel and the hard gel were prepared by mixing 160 g of ground gel with 50 mL of SSF (consisting of 40 mL of stock SSF, 0.5 mL of CaCl_2_ (0.3 M) and 9.5 mL of water).

#### 3.2.4. Human Gastric Simulator

A dynamic gastric digestion model—the Human Gastric Simulator (HGS) designed by Kong and Singh [34], —was used for the in vitro gastric digestion. A mesh bag with a pore size of 1 mm was placed inside the latex stomach chamber to mimic human gastric sieving. An enzyme solution containing 1.7 *w/v*% pepsin, 0.275 *w/v*% amano lipase A and 0.54 mmol/L CaCl_2_ was prepared to obtain pepsin and lipase activities of 1000 U/mL and 50 U/mL, and a calcium ion concentration of 0.075 mmol/L in the final gastric digestion mixture. The simulated masticated gel bolus (consisting of 160 g of ground gel and 50 mL of SSF) was warmed at 37 °C in a water bath for 2 min, and then added into the latex stomach chamber. An aliquot of 70 mL of SGF (consisting of 56 mL of stock SGF and 14 mL of enzyme solution) was also added into the stomach chamber to mimic the condition during the fasting state when the stomach contains a certain amount of gastric juice [35]. The gastric digestion time was 240 min. The temperature of the HGS was set and maintained at 37 °C by a heater and a thermostat. The gastric secretion rate was set at 2.5 mL/min [36]. The stock SGF (1.25×) and the enzyme solution were added in separately by two pumps; the secretion rates were 2 mL/min for the stock SGF and 0.5 mL/min for the enzyme solution. Gastric emptying started after 30 min because of the lag phase of solid foods [21,22]. At every 15 min, 45 mL of gastric digesta was emptied from the bottom, corresponding to an emptying rate of 3 mL/min [34].

#### 3.2.5. pH Measurement

The initial pH was defined as the pH of the simulated masticated bolus after mixing with the fasting state SGF. As access into the HGS was prevented by the simulated gastric contractions, the pH in the HGS at different digestion times was represented by the pH of the emptied gastric digesta.

#### 3.2.6. Measurement of Solid Content of Emptied Gastric Digesta

The emptied gastric digesta collected at 15 min intervals, was dried in an oven at 105 °C for 24 h to determine the dry matter content (A). In addition, a control experiment using 160 g of water instead of ground gel was carried out to determine the dry matter content of the simulated digestion fluids (i.e., SSF and SGF) retained in the emptied gastric digesta at different time points (B). The actual dry weight of the gel particles in the digesta emptied at different digestion times was determined by subtracting B from A.

#### 3.2.7. Determination of Particle Size Distribution of Emptied Gastric Digesta

A MasterSizer 2000 (Malvern Instruments Ltd., Malvern, UK) was used to measure the average diameters and the particle size distributions of the gel fragments of the emptied gastric digesta. The refractive index for the gel particles was set at 1.47. The samples were measured immediately after collection. All measurements were conducted at room temperature with three replicates. The weight-to-volume diameter *d*_4,3_ (μm) was used to denote the average gel particle size, which was calculated as follows:

(1) $$d_{4,3}=\;\sum \frac{v_{i}d_{i}}{v_{i}}$$

where *v_i_* is the volume fraction of particles with a diameter of *d_i_*.

#### 3.2.8. Determination of Oil Droplet Size Distribution

A MasterSizer 2000 was used to measure the average diameters and the particle size distributions of the oil droplets in the emptied gastric digesta. The refractive index for the oil droplets was set at 1.47. The weight-to-volume diameter *d*_4,3_ (μm) was used to denote the average oil droplet size. Aliquots of 3 mL of 5 wt% SDS solution and 20 μL of β-mercaptoethanol were added to 2 mL of emptied gastric digesta. The mixtures were then shaken overnight in a water bath at 25 °C until complete dissolution. The dissolved mixtures were used for oil droplet size measurements. All measurements were conducted at room temperature with three replicates.

#### 3.2.9. Protein Hydrolysis

Sodium dodecyl sulfate polyacrylamide gel electrophoresis (SDS-PAGE) was performed under reducing conditions to determine the protein compositions of the gastric digesta emptied at different digestion times. Immediately after being emptied from the HGS, a 20 μL of digesta sample was taken and mixed with 180 μL of electrophoresis sample buffer (0.2 M Tris–HCl buffer, pH 6.8; 40% glycerol; 2% SDS; 0.04% Coomassie Brilliant Blue G-250), 10 μL of β-mercaptoethanol was added, and then the mixture was heated in boiling water for 10 min. After cooling to room temperature, the samples were centrifuged at 4200 g for 20 min and then 10 μL of the subnatant from each sample was loaded onto tricine gels previously prepared on a Mini PROTEAN II system (Bio-Rad Laboratories, Richmond, CA, USA). The resolving gel contained 16.0 *w/v*% acrylamide, made up in Tris–HCl buffer, and the stacking gel contained 4.0 *w/v*% acrylamide, made up in Tris–HCl buffer. The electrophoresis analysis was conducted at 125 V in a cold room (4 °C) for approximately 120 min. The gel was stained for 60 min with a Coomassie Brilliant Blue R-250 solution (0.003 *w/v*% Coomassie Brilliant Blue R-250, 10% acetic acid and 20% isopropanol) under gentle shaking. The gel was firstly destained with a destaining solution of 10% acetic acid and 10% isopropanol for 1 h and then destained overnight in fresh destaining solution under gentle shaking. A Molecular Imager Gel Doc XR system (Bio-Rad Laboratories) was used to scan the gels.

#### 3.2.10. Confocal Laser Scanning Microscopy (CLSM)

A confocal laser scanning microscope (Leica, Heidelberg, Germany) was used to observe the emptied gastric digesta. Nile Red (0.1 *w/v*%) was used to stain oil (argon laser with an excitation line at 488 nm) and Fast Green (1.0 *w/v*%) was used to stain protein (He–Ne laser with an excitation line at 633 nm). An aliquot of 200 μL of emptied gastric digesta was put into a 1.5 mL Eppendorf tube and stained with 20 μL of Nile Red and 20 μL of Fast Green immediately after sample collection. Stained digesta samples were placed in an ice bath before CLSM. All stained samples were placed on concave microscope slides and covered with cover slips for CLSM.

#### 3.2.11. In Vitro Intestinal Digestion

A pH-stat (TitraLab 856; Radiometer Analytical, Villeurbanne, France) was used for in vitro intestinal digestion and to measure free fatty acids release during the in vitro intestinal digestion. The gastric digesta emptied at 60, 120 and 240 min were used for the in vitro intestinal digestion. The pH of the gastric digesta was adjusted to 7.0 using 6 M HCl/10 M NaOH immediately after emptying from the HGS. Then, 23 mL of the gastric digesta was mixed with 16.4 mL of stock SIF (1.25×) and added into the temperature-controlled chamber of the pH-stat. An aliquot of 46 mg of pancreatin from porcine pancreas (8 × USP) was dissolved in 2 mL of stock SIF, and then 46 μL of CaCl_2_ (0.3 M) was added. A total of 0.18795 g bile bovine was dissolved in 4.6 mL of Milli-Q water. The pancreatin solution and the bile bovine solution were added into the chamber at the same time and the intestinal digestion was started. The initial volume of the digestion mixture was 46 mL. The concentrations of bile bovine and CaCl_2_ in the initial digestion mixture were 10 mM and 0.3 mM, respectively. The concentration of pancreatin was 1 mg/mL in the digestion mixture, based on a trypsin activity of 100 U/mL. The intestinal digestion was carried out at 37.0 °C for 120 min with a constant stirring rate of 100 rev/min. The titration was performed with 0.05 M NaOH and the endpoint pH was set at 7.0. The quantity of free fatty acids released per mL of digestion mixture (*Q*, μmol/mL) was calculated as follows:

(2) $$Q=\frac{Volume_{NaOH}\times C_{NaOH}}{Volume_{reaction\;mixture}}$$

where *C_NaOH_* was the molar concentration of the NaOH titrant, i.e., 0.05 M. The initial lipolysis rate (μmol·mL^−1^·min^−1^) was calculated as the free fatty acids released per mL of reaction mixture per minute during the initial 2 min of reaction.

The particle size distributions of the intestinal digesta at 10, 30, 60, 90 and 120 min of intestinal digestion were measured by a MasterSizer 2000 following the method described in Section 3.2.7. The samples were measured immediately after collection. The fat content of the gastric digesta was measured by the Mojonnier method [Association of Official Agricultural Chemists (AOAC) method 989.05].

#### 3.2.12. Determination of Bioaccessibility of CAP after In Vitro Intestinal Digestion

The bioaccessibility of CAP was defined as the fraction of CAP that was released from the emulsion gel in the gastrointestinal tract after digestion and became available for intestinal absorption [37]. At the end of the intestinal digestion, part of the intestinal digesta was collected and mixed with 1mM Pefabloc solution at a volume ratio of 8:1 to terminate the enzymatic reaction. Then, the digesta was centrifuged at 17,000 g for 40 min at 4 °C. After centrifugation, the clear middle layer (i.e., the mixed micelle layer) was taken and filtered through a 0.22 μm filter. After filtration, the filtrate was considered to be the bioaccessible fraction. The concentration of CAP in the bioaccessible fraction as well as the concentration of the intestinal digestion mixture were determined by high-performance liquid chromatography. The bioaccessibility (%) of CAP was calculated as follows:

(3) $$\mathrm{Bioaccessibility}\;\left(\%\right)=\frac{\mathrm{CAP}\;\mathrm{in}\;\mathrm{bioaccessible}\;\mathrm{fraction}}{\mathrm{Total}\;\mathrm{CAP}\;\mathrm{in}\;\mathrm{digestion}\;\mathrm{mixture}}\times 100\%$$

#### 3.2.13. Quantification of Capsaicinoids by High-Performance Liquid Chromatography

The CAP contents in the mixed micelle phase (i.e., the bioaccessible fraction) and the intestinal digesta after in vitro gastrointestinal digestion of the CAP-loaded emulsion gels were quantified by reversed-phase high-performance liquid chromatography (HPLC). The chromatograph was equipped with a UV–VIS photodiode array detector (SPD-20AV, Shimadzu Corporation, Kyoto, Japan). The column was a Synergi^TM^ 4 μm Hydro-RP 80 Å liquid chromatography column with dimensions of 150 mm × 4.6 mm (Phenomenex Inc., Torrance, CA, USA). The mobile phase was composed of acetonitrile and Milli-Q water at a volume ratio of 50:50. The running temperature was set at 30 °C, with a flow rate of 1 mL/min and a sample injection volume of 5 μL. Two main compounds from CAP were detected: capsaicin and dihydrocapsaicin. The detection wavelength was set at 280 nm. CAP were extracted from the digesta samples by mixing samples and absolute ethanol at a volume ratio of 1:1. The mixtures were vortexed for 5 min, and then stored overnight at 4 °C. The next day, the mixtures were centrifuged at 10,000× *g* at 4 °C for 15 min, and then the supernatants were filtered through a 0.22 μm filter before being injected on to the chromatograph column. The quantification of capsaicin and dihydrocapsaicin was determined from a calibration curve of standard solutions of powdered CAP (capsaicin: 61.23%; dihydrocapsaicin: 31.96%; other capsaicinoids: 2.51%) in methanol.

#### 3.2.14. Statistical Analysis

Each experiment was performed in triplicates using freshly prepared samples. The results are presented as the calculated means and standard deviations. The data were analysed by one-way analysis of variance using IBM SPSS Statistics 24 software. Means were compared by Tukey tests at *P* < 0.05. Power-law fitting was performed using Origin 2017 64Bit software (OriginLab Corporation, Northampton, MA, United States). The coefficient of determination (*R*^2^, value between 0 and 1) denoted the goodness of the fit, where a value close to 1 indicated a good fit.

## 4. Conclusions

This work demonstrated the effect of gel characteristics on the in vitro gastric digestion behaviour of whey protein emulsion gels containing CAP using a human gastric simulator, and the effect of the structure of the gastric digesta on its in vitro intestinal digestion and the bioaccessibility of CAP. The results indicated that, during gastric digestion, the soft gel was disintegrated more rapidly, with the formation of small particles and free oil droplets at the end of gastric digestion. The hard gel had a much lower release of oil droplets from the protein matrix.

The hard gel also disintegrated more slowly than the soft gel during intestinal digestion because of its gel structure, except for the digesta emptied at the final stage of gastric digestion. In general, a higher fat content, larger particle size and larger oil droplet size of the gastric digesta led to a lower extent of lipid digestion. The bioaccessibility of CAP was found to be positively correlated with the extent of lipid digestion. A greater extent of lipid digestion would lead to greater release of CAP from the food matrix, and more lipolytic products would be produced and would participate in micelle formation, which would help solubilize the released CAP, and would therefore lead to a higher bioaccessibility of CAP.

This work has provided useful insights into food structural design for the delivery of lipophilic bioactive compounds; by manipulating the gel structure, the digestion behaviour in the gastrointestinal tract can be altered, which will affect the bioaccessibility of the incorporated bioactive compounds. Emulsion gel, as a model system for solid/semi-solid foods, represents many real-food products. This study on the use of gel-based delivery systems presents new possibilities for applications in the food and pharmaceutical industry (for instance, in the food industry, the structural design of spreads, sauces, yogurts, cheese, meat analogues and reformulated meat products for the delivery of bioactive compounds). Additionally, this work has provided information on how the characteristics of the gastric digesta will affect the intestinal digestion and the bioaccessibility of incorporated bioactive compounds. As emptying into the small intestine is a dynamic process; the digesta emptied at different times will differ in oil content, structure and size, which have an effect on the lipolysis behaviour during intestinal digestion. This factor should be taken into consideration to better assess the bioaccessibility of nutrients in future in vitro studies.

## Figures and Tables

**Table 1:** Initial lipolysis rate (μmol·mL^−1^·min^−1^) of emptied gastric digesta during intestinal digestion. Results are shown as mean ± standard deviation of three replicates.

	Soft Gel			Hard Gel		
	60 min	120 min	240 min	60 min	120 min	240 min
Initial lipolysis rate (μmol·mL^−1^·min^−1^)	1.05 ^a,x^ ± 0.21	1.03 ^a,x^ ± 0.38	1.48 ^a,x^ ± 0.37	0.46 ^a,y^ ± 0.06	0.64 ^ab,x^ ± 0.26	1.07 ^b,x^ ± 0.21

^a,b^ Values with different letters within the same gel type differ significantly (*P* < 0.05); ^x,y^ Values with different letters within the same gastric emptying time differ significantly (*P* < 0.05).

## Data Availability

Data available on request due to restrictions: The data presented in this study are available on request from the corresponding author. The data are not publicly available due to privacy.

## Sample Availability

Samples of the capsaicinoids are available from the authors.
